# Identification and functional analysis of a galactosyltransferase capable of cholesterol glycolipid formation in the Lyme disease spirochete *Borrelia burgdorferi*

**DOI:** 10.1371/journal.pone.0252214

**Published:** 2021-06-01

**Authors:** Petronella R. Hove, Forgivemore Magunda, Maria Angela de Mello Marques, M. Nurul Islam, Marisa R. Harton, Mary Jackson, John T. Belisle

**Affiliations:** Department of Microbiology, Immunology and Pathology, Colorado State University, Fort Collins, United States of America; University of Kentucky College of Medicine, UNITED STATES

## Abstract

*Borrelia burgdorferi (Bb)*, the etiological agent of Lyme disease, produces a series of simple glycolipids where diacylglycerol and cholesterol serve as the precursor. The cholesterol-based glycolipids, cholesteryl 6-*O*-acyl-β-D-galactopyranoside (ACGal) and cholesteryl-β-D-galactopyranoside (CGal) are immunogenic and proposed to contribute to the pathogenesis of Lyme disease. Detailed studies of CGal and ACGal in *Bb* have been hampered by a lack of knowledge of their underlying biosynthetic processes. The genome of *Bb* encodes four putative glycosyltransferases, and only one of these, BB0572, was predicted to be an inverting family 2 glycosyltransferase (GT2 enzyme) capable of using UDP-galactose as a substrate and forming a β-glycosidic bond. Comparison of the 42 kDa BB0572 amino acid sequence from *Bb* with other *Borrelia* spp demonstrates that this protein is highly conserved. To establish BB0572 as the galactosyltransferase capable of cholesterol glycolipid formation in *Bb*, the protein was produced as a recombinant product in *Escherichia coli* and tested in a cell-free assay with ^14^C-cholesterol and UDP-galactose as the substrates. This experiment resulted in a radiolabeled lipid that migrated with the cholesterol glycolipid standard of CGal when evaluated by thin layer chromatography. Additionally, mutation in the predicted active site of BB0572 resulted in a recombinant protein that was unable to catalyze the formation of the cholesterol glycolipid. These data characterize BB0572 as a putative cholesterol galactosyltransferase. This provides the first step in understanding how *Bb* cholesterol glycolipids are formed and will allow investigations into their involvement in pathogen transmission and disease development.

## Introduction

*Borrelia burgdorferi* (*Bb*) is the causative agent of Lyme disease (LD). This disease is transmitted when a mammalian host is fed upon by an *Ixodes* tick infected with *Bb* and is the most common tick-borne disease in North America [1,2]. The inflammatory immune response induced by *Bb* can result in a multisystem disease characterized by damage in various organs including the brain, central nervous system (CNS), heart, eyes, skeleton and joints [1]. The surface structures of the *Bb* spirochete contribute to the subsequent immune response that follows after infection [3]. The outer membrane structure of *Bb* is atypical of most Gram-negative bacteria as it is composed of phosphatidylcholine, phosphatidylglycerol and multiple lipoproteins, but lacks the classical Gram-negative lipopolysaccharide or lipooligosaccharide [4]. This bacterium, however, produces several glycolipids composed of a single galactose residue that modifies either diacylglycerol or cholesterol [5] to form monogalactosyl diacylglycerol (MGal) [6], cholesteryl β-D-galactopyranoside (CGal) and cholesteryl 6-*O*-acyl-β-D-galactopyranoside (ACGal) respectively. The cholesterol-based glycolipids are formed by the use of host-derived cholesterol and have been shown to be immunogenic, as well as possibly contribute to Lyme disease pathogenesis [7,8]. These cholesterol-glycolipids constitute a significant portion, 45% [9], of the total lipid from *in vitro* grown spirochetes suggesting that they are important in the physiology of *Bb*.

*Bb* has a reduced genome and thus an extremely limited biosynthetic capacity. Approximately, 100 of the 800 *Bb* chromosomal genes encoding proteins are predicted or known to be involved in the formation of biosynthetic intermediates and end products [10]. This characteristic results in *Bb* being highly dependent on host metabolites to feed critical metabolic pathways, including those that lead to the synthesis of lipids [11]. Pathogenic bacteria, including *Bb*, do not produce cholesterol, but are able to acquire it from their hosts [12,13]. A limited number of bacterial pathogens have been shown to possess the ability to modify cholesterol with carbohydrate residues via endogenous glycosyltransferases [14,15]. The only other characterized bacterial glycosyltransferase shown to produce a cholesteryl-glycoside is the GT4 family glucosyltransferase (HP0421) of *Helicobacter pylori* [16]. This protein catalyzes the transfer of glucose to cholesterol resulting in an α-glycosidic bond. Glycosyltransferases are grouped in families and share homology based on the stereochemistry of reaction products and the substrates used [16]. Thus, limited homology would exist between HP0421 and the protein of *Bb* that catalyzes the formation of the β-glycosidic bond of its cholesterol glycolipids. A search for glycosyltransferase candidates capable of forming such a bond in the CAZy database [17] led to the identification of a putative GT2 enzyme, BB0572. Following recombinant production of this protein and cell free assays, BB0572, a protein consisting of 358 amino acids and possessing a glycosyltransferase domain in the N-terminal region was demonstrated as an enzyme capable of synthesizing the cholesterol glycolipid moiety in *Bb*.

## Materials and methods

### *Borrelia burgdorferi* culture conditions

The *Bb* strain B31-A3 passage 3 strain used was a kind gift from Philip Stewart, Rocky Mountain Laboratories, NIAID, NIH, Hamilton, MT. Cells were cultured in Barbour-Stoenner-Kelly II (BSK-II) medium supplemented with 6% rabbit serum (Cedarlane Laboratories, Burlington, NC) at 35°C in 2.5% CO_2_. Cells were grown to mid log phase ~4–6 x10^7^ before being subcultured to desired starting density. Cell densities and growth phases were monitored by dark-field microscopy and enumerated using the 2-Chip Hemocytometer (Bulldog Bio Inc., Portsmouth, NH).

### Cloning and expression of recombinant *bb0572*

The gene *bb0572* was synthesized by GeneArt Gene Synthesis (Thermo Fisher Scientific, Waltham, MA) with *Nde*I and *Xho*I sites at the 5`and 3`ends, respectively, and inserted into pMA-RQ (Thermo Fisher Scientific) to form pPHM006. The *Nde*I allowed for an ATG start site and the *Xho*I site was after the stop codon. The gene sequence was confirmed and cloned into the *Nde*I and *Xho*I restriction sites of pET28a (MiliporeSigma, Burlington, MA) resulting in plasmid pPHM007. A mutated form of *bb0572* lacking the codons for three predicted active sites (CFF, DGD, and I) was generated using GeneArt Mutagenesis (Thermo Fisher Scientific). The synthetic gene (*bb0572 ΔCFF/DGD/I*) was inserted into pMA-T to form pHM008 and subsequently cloned into pET28a to form pPHM009. The wild type and mutated *bb0572* were expressed in *E*. *coli* BL21-CodonPlus (DE3)-RIPL (Agilent Technologies, Santa Clara, CA) transformed with pMH007 and pMH009, respectively. The recombinant *E*. *coli* were grown in LB at 37°C to a cell density of 1x10^8^ and gene expression was induced with 1 mM IPTG at 37°C for 3 h.

### Detection of recombinant proteins

#### Western blot analysis

Expression of *bb0572* constructs in *E*. *coli* BL21-CodonPlus (DE3)-RIPL was confirmed by Western blot. Following induction, lysates were loaded onto a NuPAGE 4–12% (Invitrogen; Carlsbad, CA) and run at 200 V for 30 min. Proteins were electrotransferred to nitrocellulose membranes and blocked for 1 h at room temperature with 1% BSA in TBST (Tris-buffered saline, 0.1% Tween 20). Primary mouse 6x His-Tag monoclonal antibody (Invitrogen) was diluted in 1% BSA TBST and incubated overnight at 4°C. Washes were performed with 1 × TBST before the addition of horseradish peroxidase conjugated to goat anti-mouse secondary antibody (Invitrogen) for 1 h at room temperature. Blots were developed using Clarity Western ECL Substrate (Bio-Rad, Hercules, CA) and imaging was done using ChemiDoc™ Gel Imaging System (Bio-Rad).

#### LC-MS/MS analyses

Whole cell lysate (5 μg protein) was resolved by SDS-PAGE using NuPAGE 4–12% (Invitrogen) gels and stained with Coomassie Brilliant Blue (Thermo Fisher Scientific). Gel slices of proteins that migrated at ~25, 37 and 75 kDa were excised and subjected to in-gel trypsin digestions. Data acquisition of resulting peptides by LC-MS/MS was performed on an Orbitrap Velos Pro mass spectrometer (Thermo Fisher Scientific) and data analyses were carried out as described in Supplementary Material (S6 File). The LC-MS/MS analyses were conducted by the Analytical Resources Core: Bioanalysis and Omics facility at Colorado State University.

### Expression of *bb0572* by *Bb*

To assess the expression of *bb0572*, the spirochete was grown to mid-log phase, subcultured at 1x10^3^ cells per mL and grown to a density of 1x10^6^ cells per mL. Aliquots (10 mL) of the culture were harvested for RNA extraction using the Direct-zol RNA Miniprep Plus (Zymo, Irvine, CA) followed by DNase-treatment using the RQ1 RNase-Free DNase (Promega, Madison, WI). The remaining aliquots continued incubation and were harvested for RNA isolation at 6, 12, 18, 24, 30 and 36 h. Cell densities were also determined concurrently at stated time points by dark-field microscopy. RT-qPCR was performed using the Luna Universal One-Step RT-qPCR Kit, (New England Biolabs, Ipswich, MA) with a LightCycler® 480 (Roche Life Science, Penzberg, Germany) and the following cycling conditions: Reverse transcription at 55°C for 10 min, initial denaturation at 95°C for 1 min, and 40 cycles of denaturation at 95°C for 10 sec, followed by extension and product detection at 60°C for 1 min. Expression was normalized to the *flaB* [18,19] housekeeping gene using previously published primers [3] (**Table 1**). We used the 2 delta delta method [20] to analyze gene expression based on normalization with the *flaB* as the house keeping gene relative to time zero (when cells reached early exponential). The average of the Ct values of *flaB* and the target gene BB0572 were determined. The experiment Ct values were those obtained after amplification of RNA on samples harvested at 6, 12, 18, 24, 30 and 36h, the control Ct values or baseline in this experiment values obtained from cells harvested at time 0. The fold change increase is depicting increase of normalized expression compared to the initial time of cell harvest.

**Table 1:** Primers used in this study.

Primer	Sequence (5`-3`)	Description	Reference
PHM21	GAGTTTCTGGTAAGATTAATGCTC	*flaB* Forward primer for qRT-PCR	[3]
PHM22	CATTTAAATTCCCTTCTGTTGTCTGA	*flaB* Reverse primer for qRT-PCR	[3]
PHM43	GCCTCAGAAAGTCCCTTGTC	*bb0572* Reverse primer for qRT-PCR	This study
PHM44	GGTAGTTTAGAGATAGCAG	*bb0572* Forward primer for qRT-PCR	This study

### Cell free assay for cholesteryl galactoside formation

The *E*. *coli* BL21-CodonPlus (DE3)-RIPL containing recombinant *bb0572* constructs, (pPHM007 and pPHM009) were grown in 20 mL cultures and gene expression induced as described. *B*. *burgdorferi* strain B31 was grown in 20 mL cultures to approximately 10^8^ bacteria/mL. Cells were harvested by centrifugation at 10,000 x g for 10 min, and suspended in 500 μL assay buffer [110 mM HEPES pH 8.0, 22 mM MgCl_2_ and 22 mM CHAPS; (Boehringer Mannheim, Mannheim, Germany)] [6]. Cells were lysed on ice by sonication using a 60 Sonic Dismembrator Cell Disrupter (Thermo Fisher Scientific) at an output power of 20 watts with five successive 1 sec pulses followed by a 30 sec rest. This cycle was repeated three times. Protein concentration was determined by measuring absorbance at 280 nm by spectrophotometry. A mixed micelle of 1 mM dioleoyl-phosphatidylglycerol (Avanti Polar Lipids, Alabaster, AL), 1 mM rac-1,2-dioleoylglycerol (Sigma-Aldrich, St. Louis, MO) and 0.5 μCi [26-^14^C] (Quotient Bioresearch Ltd., Cardiff, United Kingdom) cholesterol at a specific activity of 50 mCi/mmol was prepared. Specifically, lipids were solubilized in chloroform/methanol (1:1 v/v), dried by evaporation under N_2_, and solubilized to homogeneity in 20 μL assay buffer and incubated overnight at 4°C.

Aliquots (25 μL) of each cell lysate (~0.5 mg of protein) was added to 20 μL of mixed micelle suspension and incubated on ice for 30 min. Enzymatic reactions were initiated by the addition of UDP-galactose (1 mM final concentration) and incubated for 30 min at 28°C as previously described [6]. As controls the enzymatic assays were performed by addition of 1 mM GDP-mannose (Sigma-Aldrich) instead of UDP- galactose to determine substrate specificity. To confirm that the galactose residue was attached at 3C position of cholesterol, 1 unit of cholesterol oxidase, which oxidizes cholesterol to cholest-5-en-3-one was added to the reaction [21]. Boiled lysate and *E*. *coli* strain harboring an empty plasmid were also included to represent absence of enzymatic activity. Methanol/chloroform (2:1 v/v) was added to stop the reaction and extract lipid products. The lipid extract was dried under N_2_ and suspended in chloroform/methanol (2:1 v/v). The lipid extracts were resolved by thin-layer chromatography (TLC) using silica gel G60 TLC plates and chloroform/methanol (90:10 v/v) as the mobile phase. Reference non-radioactive standards were resolved concurrently and developed separately from the blot containing radioactive lipids with phosphomolybdic acid stain. Following this, blot with reference standard was matched with TLC blot to mark resolution of lipids. After development radiolabeled lipids were imaged using Azure Sapphire Biomolecular Imager (Azure Biosystems Inc, Dublin, CA). Standards of MGal, CGal were obtained from (Avanti Polar Lipids) and ACGal from purified *Bb* cell culture.

### *In silico* analyses

Carbohydrate active enzymes were searched in the Carbohydrate-Active Enzymes (CAZy; http://www.cazy.org) database by querying the organism name. Selected organism name was *Borrelia burgdorferi* B31. A standard protein blast (BLASTP, https://blast.ncbi.nlm.nih.gov/) search of BB0572 against the Lyme disease (LD) taxid (taxid: 64895) and relapsing fever (RF) spirochetes taxid (taxid:138) (**S1 Table**). Sequence alignment was performed with the Tree based Consistency Objective Function for AlignmEnt Evaluation (T-Coffee): http://tcoffee.crg.cat/apps/tcoffee/do:regular [22] using FASTA sequences from NCBI. Core/TS [23,24] was used to evaluate amino acid alignments and Boxshade (http://www.ch.embnet.org/software/BOX_form.html) was used to generate alignment images. Evolutionary analyses were conducted in MEGA X [25] were distances were computed using the Poisson correction method [26] with units of the number of amino acid substitutions per site. To predict cellular location, BB0572 protein sequence was interrogated with PSORT (http://www.psort.org/psortb/) [27], candidate localization-sites for prediction was based on Gram-negative classification, and SignalP 5.0 http://www.cbs.dtu.dk/services/SignalP/ [28].

### Statistical analysis

GraphPad Prism 8.1.2 software was used for *in-vitro* expression data analysis. The one-way analysis of variance (ANOVA) was used followed by the all-pairwise multiple-comparison procedure (Tukey multiple comparison test) if there were significant differences among treatment means (p < 0.05).

## Results

### Bioinformatic identification of BB0572 as a putative galactosyltransferase

**Fig 1:**
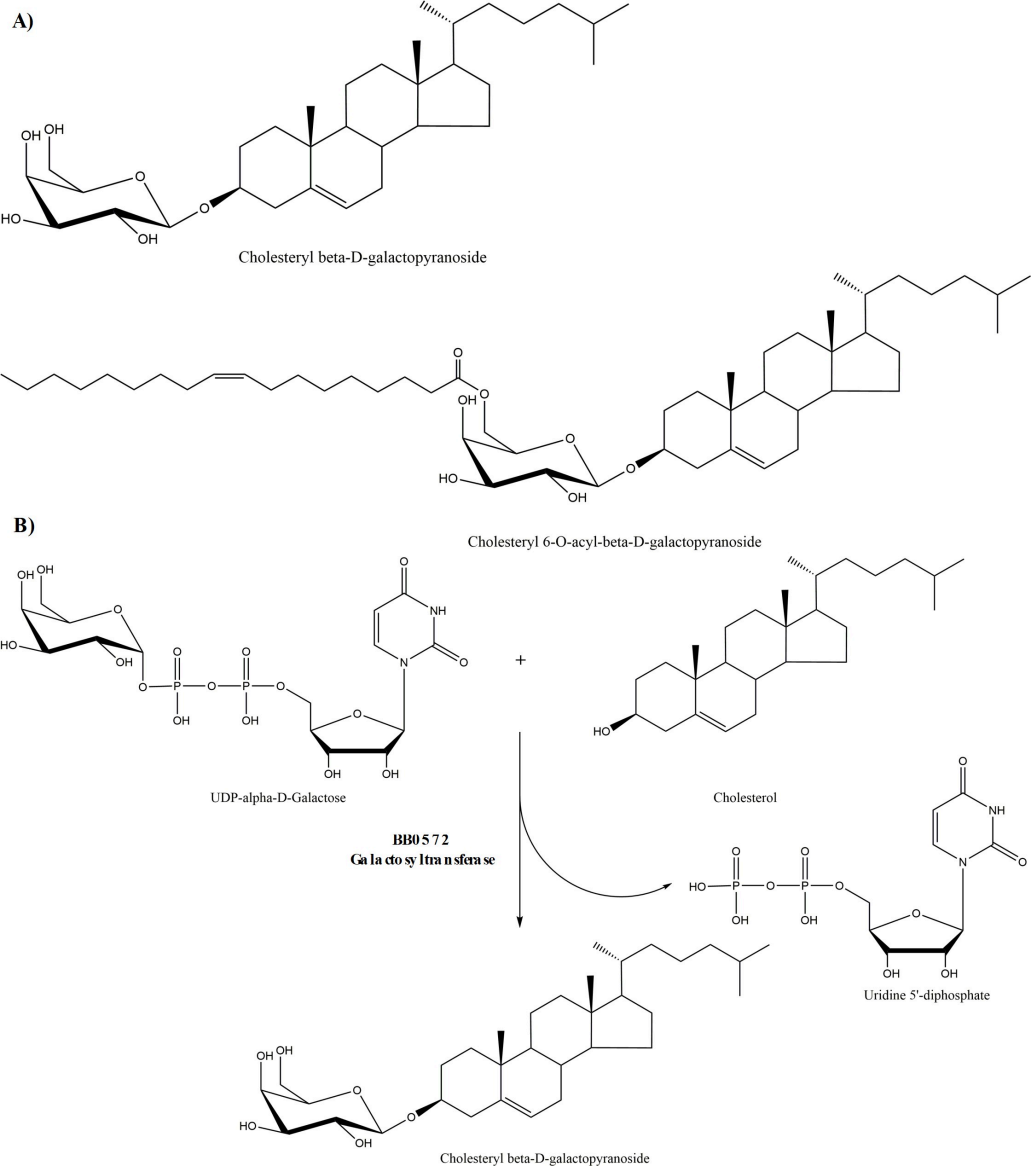
The cholesterol glycolipids of *Borrelia burgdorferi* and predicted reaction. A. Chemical structure of cholesteryl-β-D-galacto-pyranoside (CGal); and cholesteryl 6-O-acyl-β-D-galactopyranoside (ACGal) **B**. Predicted reaction showing UDP-alpha-galactose as the sugar donor and cholesterol as the acceptor molecule to yield cholesteryl β-D-galactopyranoside (CGal) which possess a galactosyl residue with its anomeric bond in the *β-*configuration.

**Fig 2:**
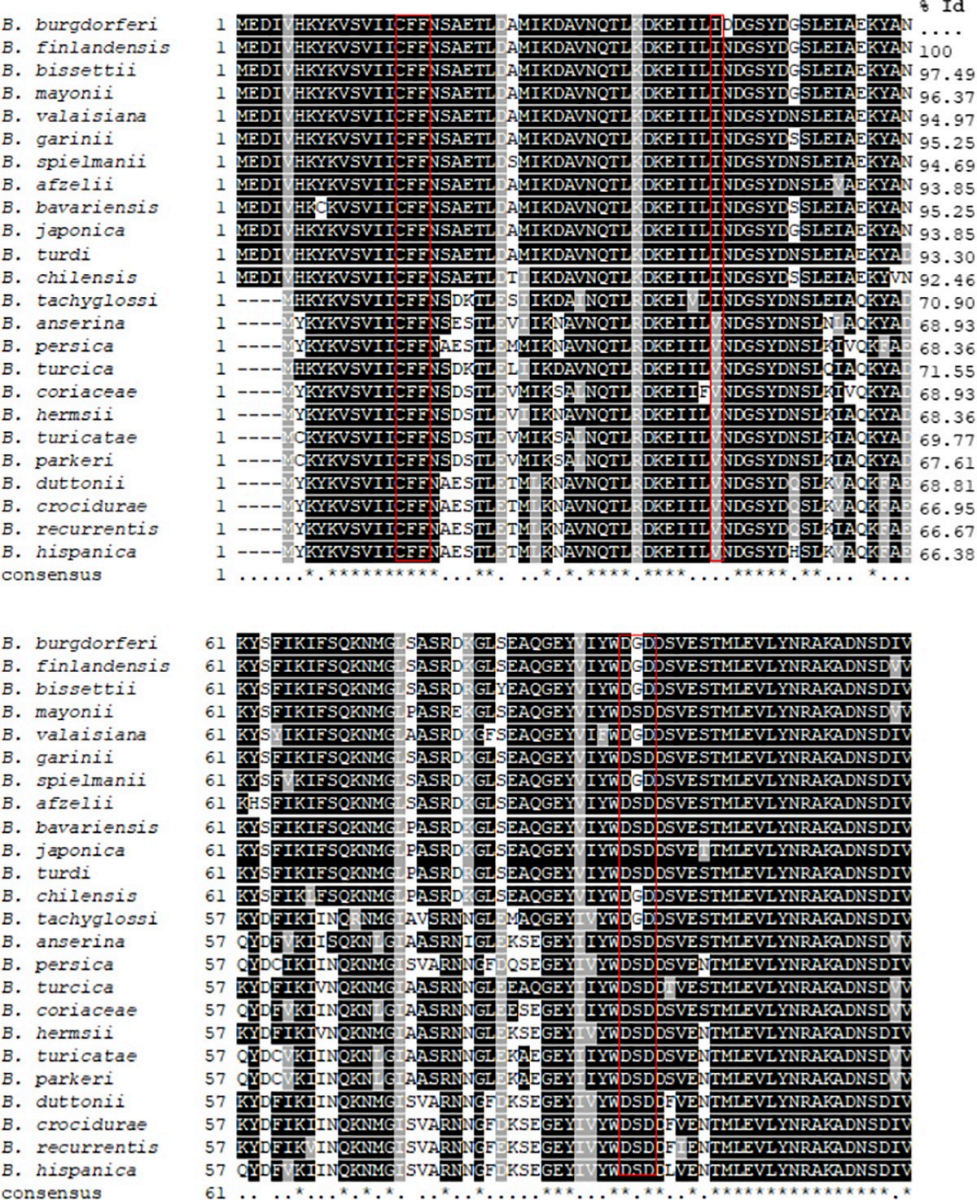
Multiple sequence alignment of the BB0572 sequence from *Borrelia burgdorferi* against Lyme disease and relapsing fever causing spirochetes. Red box indicates the active motifs of the GT2 glycosyltransferase enzyme. A star indicates positions which have a single, fully conserved residue. A period (.) indicates conservation between groups of weakly similar properties.

The cholesterol glycolipids of *Bb*, possess a galactosyl residue with its anomeric bond in the *β-*configuration [5,8] **Fig 1A**. This structure along with UDP-Gal as the presumptive sugar donor indicates that formation requires a galactosyltransferase of the GT2 family of inverting glycosyltransferases [29], predicted reaction depicted in **Fig 1B**. The CAZy database catalogs known and presumptive glycosyltransferases to 111 distinct sequence-based GT families [30]. Interrogation of CAZy for glycosyltransferases produced by *Borrelia* spp yielded 4 predicted glycosyltransferases. Only one of these, however, was of the GT2 family. This gene product in *Bb* strain B31 was designated BB0572 (glycosyl transferase (lgtD)) position 585080–586156 on *Bb* chromosome, length: 1077 bp. (358 amino acids) [10], confirming an earlier report by Ӧstberg *et al* [6]. The amino acid sequence of BB0572 possesses a region (amino acid 11–182) that is conserved in the GT2 glycosyltransferase family [31]. Within this region were predicted active site motifs, CFF (amino acid 15–17), I (amino acid 43) and DGD (amino acid 95–97) which corresponds roughly to the nucleotide-binding domain that is defined by the DxD motif **Fig 2** [29].

A BLASTP search of the BB0572 sequence against the annotated genomes of other *Borrelia* spp. of the Lyme disease taxid (taxid: 64895) and relapsing fever spirochetes taxid (taxid:138) **(S1 Table)** demonstrated homologues of BB0572 in all species [32]. Of note among the LD causing spirochetes were *Borrelia afzelii* (Europe, Asia), and *Borrelia garinii* (Europe, Asia) [33] and the newly discovered *Borrelia mayonii* [34]. The amino acid alignment of BB0572 to the homologues in other *Borrelia* spp (**Fig 2)** revealed the protein was highly conserved among the *Borrelia* spp (T Coffee score of 998). Amino acids forming the putative glycosyltransferase active site motifs (CFF and DGD) were conserved in all the proteins of all species analyzed. The isoleucine (I) at position 43 of BB0572 was also predicted to be a conserved active site residue of the GT2 family. This amino acid was conserved in the Lyme disease spirochetes but was substituted for valine (V) in the relapsing fever spirochetes. The conservation of sequence was high within each taxid. The Lyme disease and relapsing fever spirochetes taxids yielded proteins with 91 to 100% and 96 to 100% identity, respectively. In general, the protein sequence was found to be four amino acids shorter at the N-terminus in the relapsing fever spirochetes compared to the Lyme disease spirochetes (**Fig 2)**. The evolutionary relationship of BB0572 and its homologues among *Borrelia* spp is represented in (**S1 Fig)**, with clades separating based on LD and RF pathogens.

The putative subcellular localization of BB0572 was evaluated using SignalP 5.0 http://www.cbs.dtu.dk/services/SignalP/ [28] and PSORTb (http://www.psort.org/psortb/) [27]). SignalP did not predict the presence of a signal peptide. Thus, BB0572 was predicted not be translocated. PSORTb was used to predict the cellular location of BB0572 (**S1 File)** as a cytoplasmic membrane protein. However, this was based on a SubCellular Localization (SCL)-BLAST match to a hyaluronan synthase of *Pasteurella multolcida*. The potential for cytoplasmic transmembrane domain was interrogated via TMPred (https://embnet.vital-it.ch/software/TMPRED_form.html). This indicated weak transmembrane domains at the C-terminus of BB0572. Overall, bioinformatic analyses did not provide a strong indication of the subcellular location for this protein, but a cytoplasmic or cytoplasmic membrane localization [35,36] consistent with the use of a nucleotide sugar as the donor substrate.

### Recombinant expression of BB0572 and galactosyltransferase activity

**Fig 3:**
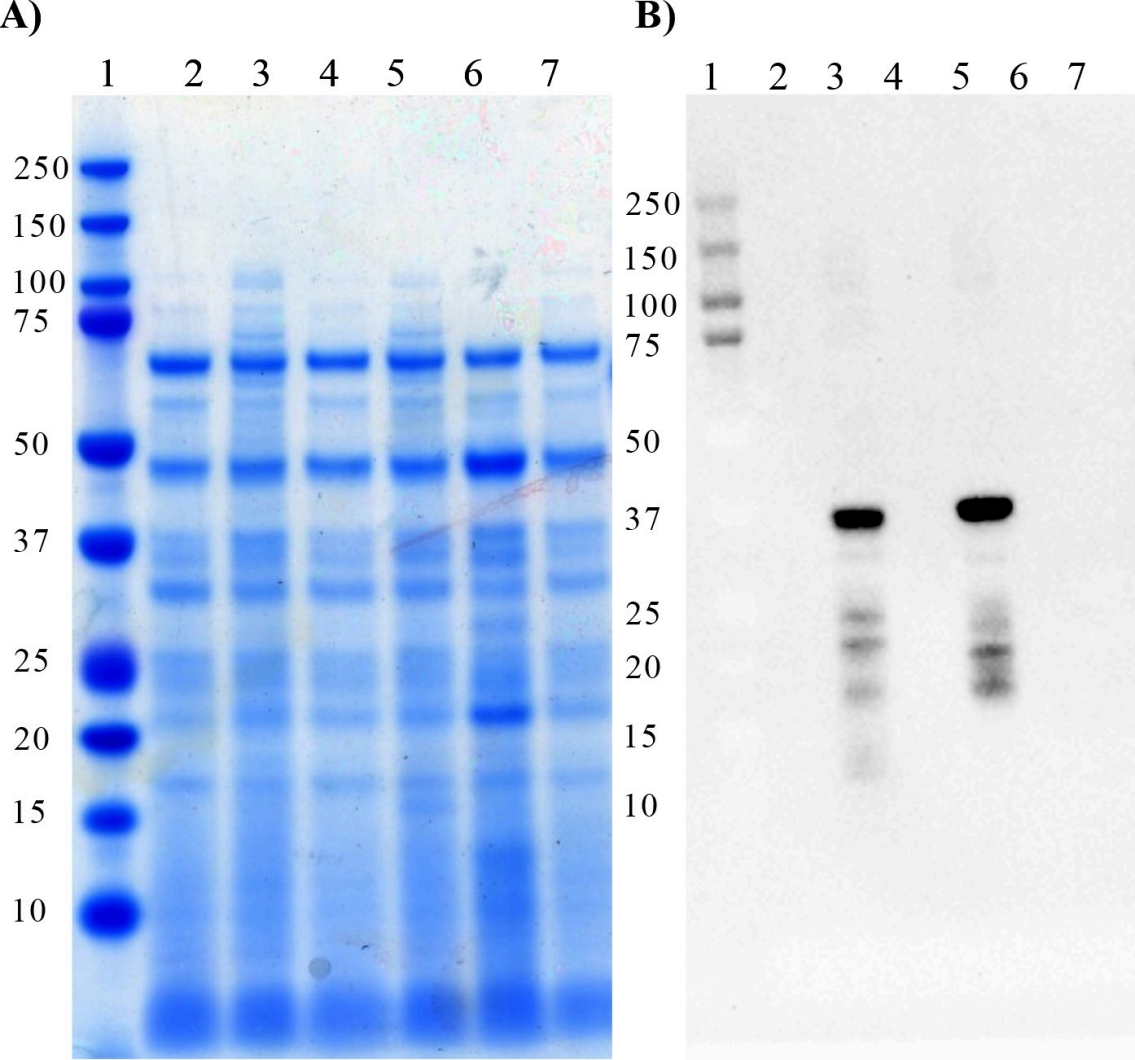
Verification of expression of *bb0572* in *E coli*. Coomassie stained SDS-PAGE gel (**A**) and anti-His tag Western blot (**B**) of whole cell lysate from uninduced (lanes (2, 4 and 6) and IPTG induced (lanes 3, 5 and 7) cultures of *E*. *coli* expressing the WT *bb0572* (lane 2 and 3), mutated *bb0572 ΔCFF/DGD/I* (lane 4 and 5) or possessing the empty pET28a vector (lane 6 and 7). Lane 1(ladder).

To maximize protein production, the gene encoding BB0572 was synthesized with optimal codon usage and expression in *E*. *coli*. The optimized *bb0572* gene and *bb0572* ΔCFF/DGD/I mutant were expressed in BL21-CodonPlus (DE3)-RIPL. Initial analysis of the whole cell lysates by SDS-PAGE and Coomassie staining did not reveal a dominant difference between the induced and uninduced cells in the 37 kDa range (expected size 42KDa) for the recombinant BB0572 or mutated BB0572 **(Fig 3A**). However, following Western blot using an anti-His tag probe, a dominant reactive band was observed at the 37KDa weight marker kDa, and less reactive bands at 25 kDa and 75 kDa (**Fig 3B**). These latter two products were potentially a degradation product and a dimer, respectively, of the recombinant BB0572 fusion proteins. To verify production of recombinant BB0572, LC-MS/MS was performed on tryptic digests of gel bands at 37, 25 and 75 kDa range. Analysis of proteins migrating at 37 kDa resulted in the identification 82 proteins. The proteins with the greatest abundance based on normalized spectral abundance factor (NSAF) were the 39 kDa OmpF and 36 kDa GapA of *E*. *coli*, followed by the recombinant BB0572. The MS/MS data revealed the detection of 392 total spectra representing 14 unique peptides and ~35% amino acid sequence coverage for recombinant BB0572 (**S2 and S3 Files**). The proteins migrating at 25 kDa and 75 kDa also contained presence of recombinant BB0572, but at greatly decreased abundance as measured by NSAF and the percent amino acid sequence coverage **(S2 Fig)**. These data confirmed that products from recombinant *E*. *coli* and detected by Western blot were the BB0572 constructs and that the less abundant 25 kDa and 75 kDa products were degraded products of the recombinant BB0572 fusion proteins **(S3 File)**. This also serves to explain the extra bands observed by Western blot **(Fig 3)**.

### Expression of *bb0572* in *Bb* and cell free assay of cholesteryl galactoside synthesis

**Fig 4:**
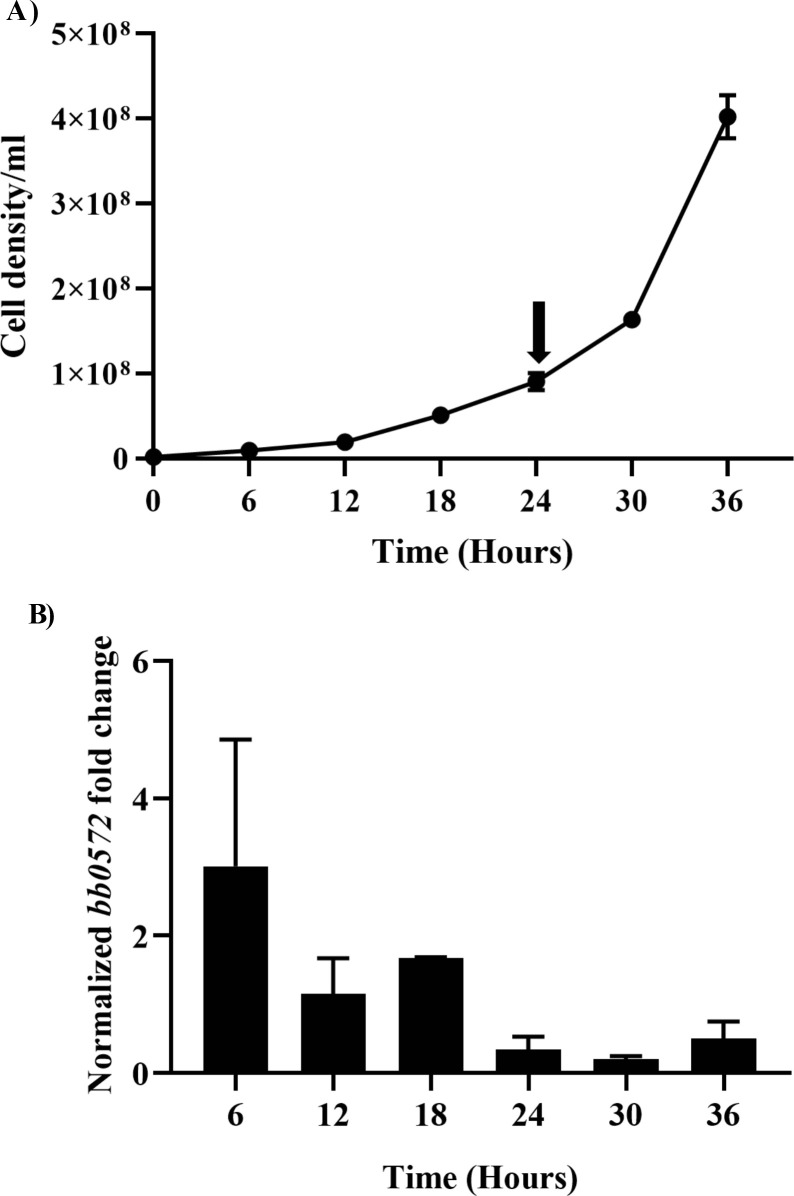
Cell density and kinetics of *bb0572* expression in *Borrelia burgdorferi* cultured *in vitro*. *Bb* cells at early exponential phase (1x10^6^) were cultured and harvested at 6, 12, 18, 24, 30 and 36 h to determine expression of *bb0572*. **A**. Cell densities were monitored by dark-field microscopy every six hours, corresponding to cell harvest for RNA extraction for expression analysis. **B**. Gene expression of *bb0572* was normalized with the *flaB* as the house keeping gene. Fold change represents change in expression of *bb0572* between 6 to 36 h relative to time zero (baseline). No significant differences in expression were noted during selected time intervals. To ensure adequate number of cells, culture with cell density of (1 x10^8^ cells/ml) was used in cell-free assays (black arrow).

To determine whether *Bb* actively expressed *bb0572*, *Bb* cells were subcultured to 1x10^3^ and left to grow to a cell density of 1x10^6^ (~ early exponential phase). After reaching 1x10^6^ cells, an aliquot of cells representing baseline expression were harvested. Cells were left to incubate and were harvested at 6, 12, 18, 24, 30 and 36 h (**Fig 4 and S4 File**) and cell densities determined concurrently. Expression of *bb0572* steadily declined from 6 h and remained fairly constant during the study interval (9 x10^6^ cells/ml (6 h) and 4.5x10^8^ cells/ml (36 h). However, this may not reflect protein levels of BB0572 in the absence of Western blot data. No statistical differences were detected when gene expression was compared across time points studied (p> 0.05). To ensure adequate cells numbers, whole cell extracts of spirochetes grown to 1 x10^8^ cells/ml were used in subsequent cell-free assays.

**Fig 5:**
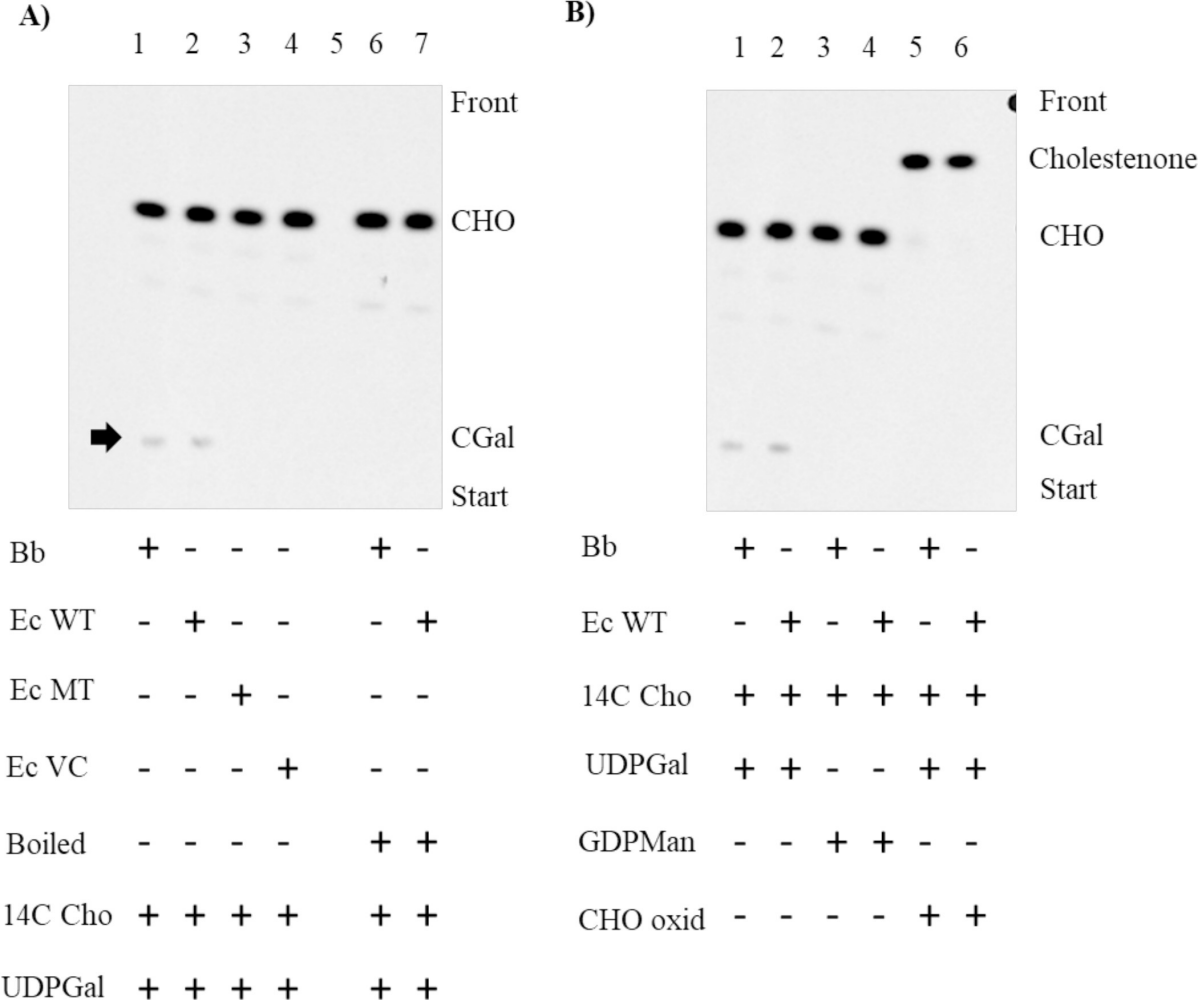
TLC of lipids from cell free assays for the enzymatic incorporation of [26- ^14^C] cholesterol into cholesteryl-β-D-galacto-pyranoside (CGal). A. The formation of cholesteryl-β-D-galacto-pyranoside (CGal) (arrow) in whole cell lysates of *Bb* and recombinant *E*. *coli expressing WT bb0572* (lane 1 and 2). As expected, CGal was not formed by the whole cell lysate of *E*. *coli* expressing mutant *bb0572 ΔCFF/I/DGD* (lane 3). Lane 4 is the empty expression vector control, and lane 6 and 7 are from boiled whole cell lysates of *Bb* and recombinant *E*. *coli expressing WT bb0572*, respectively. Note lane 5 was not used. **B.** Substrate controls: *Bb* and *E*. *coli WT bb0572* whole cell lysates with UDP-Gal (lane 1 and 2); GDP-Man (lanes 3 and 4), or cholesterol oxidase treated [26- ^14^C] cholesterol with UDP-Gal (lane 5 and 6) as substrate. CHO, cholesterol; ACGal, cholesteryl 6-*O*-acyl-β-D-galactopyranoside and MGal, monogalactosyl diacylglycerol. Ec WT (*Escherichia coli* wild type), Ec MT (*E*.*coli* mutant—*bb0572* ΔCFF/DGD/I) and Ec VC- *E*.*coli* vector control).

To test the primary hypothesis that BB0572 possessed the galactosyltransferase activity responsible for formation of CGal, a mixed micelle preparation containing 0.5μl radiolabeled [26-^14^C] cholesterol (specific activity, 50 mCi/mmol) (0.5 μCi) was added to whole cell lysates of recombinant *E*. *coli* strains and *Bb* along with UDP-Gal to initiate the enzymatic reaction. The biosynthesis of CGal in the *Bb* lysate was observed by TLC (**Fig 5A lane 1**) at expected retention factor (Rf) of this product based on co-migration of a radiolabeled product with the CGal chemical standard. The same radiolabeled lipid was also observed in recombinant *E*. *coli* producing WT BB0572 (**Fig 5A lane 2**). In contrast, this lipid product was absent from recombinant *E*. *coli* producing the ΔCFF/DGD/I mutant of BB0572 (**Fig 5A lane 3**) or *E*. *coli* possessing the empty pET28a vector (**Fig 5B, lane 4**). Also included were standards for ACGal and monogalactosyl diacylglycerol (MGal) [6], the glycolipid of *Bb* that contains no cholesterol. There was no detection of ACGal and MGal at expected Rf of these products based on co-migration with a ACGal purified from *Bb* and by use of a chemical standard for MGal. As controls the *Bb* and recombinant *E*. *coli* producing BB0572 lysates were boiled prior to initiation of the enzymatic reaction (**Fig 5A lane 6 and 7)**. This prevented the formation of CGal. To confirm substrate specificity the *Bb* and recombinant *E*. *coli* producing BB0572 lysates were incubated with either UDP-galactose **(Fig 5B lane 1 and 2)** or GDP-mannose (**Fig 5B lane 3 and 4**). No product was formed when GDP-mannose was used. Cholesterol oxidase converts cholesterol to cholestenone, by converting the hydroxyl residue at the 3C position of cholesterol to a ketone; the 3C position of cholesterol is the site of glycosylation in CGal. When [26-^14^C] cholesterol was treated with cholesterol oxidase prior to initiation of the galactosyltransferase assay, all of the cholesterol was converted to cholestenone based on migration of band above cholesterol and as previously shown [37]. In addition, the ability of the *Bb* and recombinant *E*. *coli* producing BB0572 lysates to form CGal was lost (**Fig 5B lane 5 and 6)**. Also observed were faint bands between cholesterol and the CGal products. These were ruled as nonspecific as they appeared in all lanes (**Fig 5A and 5B**).

Findings from the recombinant expression and cell free assay of cholesteryl galactoside synthesis, provided evidence that BB0572 is the putative galactosyltransferase responsible for the formation of CGal in *Bb*. Analysis did not however yield a band corresponding to ACGal.

## Discussion

The goal of this study was to identify the galactosyltransferase responsible for the synthesis of *Bb* cholesterol glycolipids. These products are formed in *Bb* through acquisition of host cholesterol during infection or in vitro growth [12]. The enzymes of eukaryotes and prokaryotes that catalyze the transfer of glycosyl residues from nucleotide-sugar or polyprenyl-phospho-sugar donors onto a variety of acceptors comprise multiple glycosyltransferase families [38]. Our bioinformatic search of the annotated *Bb* genome yielded only one predicted GT2 inverting enzyme with a common GT-A type structural fold, BB0572 (glycosyl transferase (lgtD)) position 585080–586156 on *Bb* chromosome, length: 1077 bp. (358 amino acids). This confirmed a previous report by Ӧstberg *et al* that predicted BB0572 as the galactosyltransferase for *Bb* cholesterol glycolipids [6]. The GT2 family is represented by more than 3,500 sequences, originating from animal, plant, yeast, and bacterial species, with at least 12 distinct GT2 functions having been characterized [29]. In this study we functionally evaluated a member of this enzyme family. Findings from the recombinant expression and cell free assay provided preliminary evidence that BB0572 is a functional galactosyltransferase catalyzing the formation of the glycosidic bond between cholesterol and a galactose sugar moiety, forming CGal in *Bb*. From these results and our bioinformatic analyses we hypothesize that BB0527 is the only galactosyltransferase responsible for synthesis of CGal and ACGal. However, further studies involving the mutagenesis of *bb0572* in *Bb* are required to test this hypothesis. It is noted that previous studies based on signature-tagged mutagenesis [39] suggests that *bb0572* might be essential for in vitro growth of *Bb*. Thus, evaluation of the biological significance of *bb0572* warrants further investigation.

Our cell free assays did not yield a radiolabeled lipid corresponding to ACGal. This was likely due to the low yield of CGal and that formation of ACGal likely requires addition of the appropriate acyl-donor to the cell free assay. Further investigation is required to identify the acyltransferase responsible for acylation of CGal and the appropriate acyl-donor. At this point, the acylation of UDP-galactose prior to the donation of the sugar to cholesterol cannot be ruled-out. However, this is very unlikely, as bioinformatic analyses indicated BB0572 as the only galactosyltransferase of *Bb* that will form the β-glycosidic bond found in CGal and ACGal, and the transfer of an acylated galactose from UDP to cholesterol would require a galactosyltransferase other than BB0572.

Glycosyltransferases, in general, show great structural diversity ranging from as small as 200 amino acids to as large as 1500 amino acids long [40]. In both RF and LD spirochetes the sequence of the predicted galactosyltransferase is highly conserved. This is not unexpected as classification of glycosyltransferases is based on amino acid sequences [41]. The length variation between the LD and RF spirochetes BB0572 homologues ranged from 354 to 358 amino acids. Of the variable sequences the main difference occurs in the N terminus were the BB0572 homologues of LD spirochetes possess additional four amino acid residues, MEDI. Absence of these four amino acids does not seem to alter the prediction of enzyme location for the RF spirochetes. We also found that the BB0572 of *Bb* was predicted to be membrane bound and so was the RF spirochete homologue. Additional variable regions are noted between the BB0572 homologues of the LD and RF spirochetes. However, further investigation is required to determine whether functional differences exist between the galactosyltransferase activity in these two groups of pathogens.

Cholesterol presence in the cell membrane is a unique feature in bacteria as they rarely synthesize this molecule [42]. A few bacteria (e.g. *Micrococcus lysodeikticus*, *Bacillus megaterium*, *Proteus mirabilis* [42–44] *Mycoplasma*, *Helicobacter* and *Borrelia* species) have been reported to acquire cholesterol from the environment as a requirement for growth [42]. Among these *Helicobacter pylori* [14,16,44] *Mycoplasma gallinarum*, [45] *Acholeplasma axanthum* [15] and *Borrelia* species conjugate cholesterol to carbohydrates through the action of glycosyltransferases, to form cholesterol glycolipids. These structures are however abundant widespread membrane lipids, occurring in all plants, several algae [46,47], some fungi [48–50], slime molds [51,52] and animals [53–56].

The cholesterol glycolipids of *Borrelia* spp. were first reported in 1978 for *B*. *hermisii* a RF spirochete [57]; later in the LD spirochetes, and gained importance as immunogenic structures [5,7–9,58,59]. The LD cholesterol glycolipids contain galactose; however, a single report on *B*. *hermsii* used radiolabeled glucose for metabolic labeling of the cholesterol glycolipids [8]. This has resulted in this lipid being identified as a glucosyl cholesterol, but the structure of the *B*. *hermsii* cholesterol glycolipid has not been elucidated and it is possible that metabolic labeling with glucose could give rise to galactose. Interestingly cholesterol glycosides from the other bacteria, *H*. *pylori* [14,16,44] *M*. *gallinarum*, [45] *A*. *axanthum* [15] contain glucose as the carbohydrate, making the cholesterol glycolipids of *Bb* unique. This might have a bearing on function or on disease pathogenesis. In *Helicobacter* cholesteryl glucosides play an important role in colonization of mice and immune evasion [60] as well as virulence and contributes to intrinsic antibiotic resistance [61]. In *Bb* there is evidence that cholesterol glycolipids form lipid rafts [62–66] similar to those found in eukaryotes. However there is no record of a direct role of these lipids in the pathogenesis of LD, except that an immune response is formed against ACGal [9,58]. With the identification of BB0572 as the putative enzyme for galactosyltransferase catalyzing the formation of *Bb* cholesterol glycolipids, it will now be possible to generate studies to decipher the role of these lipids in *Bb* physiology and pathogenesis. In addition, more information is needed to understand how BB0572 is expressed during different phases of growth as it is currently unknown if this enzyme and its products are an essential requirement for *Bb* growth.

## Supporting information

S1 Fig Evolutionary relationships of taxa for BB0572. Evolutionary analyses were conducted in MEGA X using the Minimum Evolution method [1]. The analysis used 23 amino acid sequences and a total of 359 positions in the final dataset. The tree shown has a sum of branch length of 1.25967424. The branch lengths represent the units of evolutionary distances that infer the phylogenetic relationship calculated using the Poisson correction method. Distances indicate the number of amino acid substitutions per site. The Close-Neighbor-Interchange (CNI) algorithm was used to search the ME tree at a search level of 1.
(TIFF)

S2 Fig Amino acid coverage for the Identification of BB0572. LC-MS/MS was performed on tryptic digests of gel bands at 37, 75 and 25 kDa bands observed by SDS-PAGE and Western blotting. The amino acid sequence shown is for BB0572 and the yellow highlighted regions represent the peptide sequences identified by LC-MS/MS.
(TIF)

S1 File Output for the putative subcellular localization of BB0572 as determined by PSORTb. (PDF)

S2 File Identified proteins by LC-MS/MS. (XLSX)

S3 File Peptide and spectra count for BB0572 in the 37, 25 and 75 kDa samples. (XLSX)

S4 File RT-qPCR supporting information. (PDF)

S5 File Raw images. (PDF)

S6 File Mass spectrometry analysis. (DOCX)

S1 Table Selected *Borrelia* species for protein alignment and evolutionary analyses. (DOCX)
