# Supplementary figures and images for: Identification and functional analysis of a galactosyltransferase capable of cholesterol glycolipid formation in the Lyme disease spirochete Borrelia burgdorferi
Source: PLoS One. 2021 Jun 1;16(6):e0252214. doi: 10.1371/journal.pone.0252214 (PMC8168883; doi:10.1371/journal.pone.0252214)

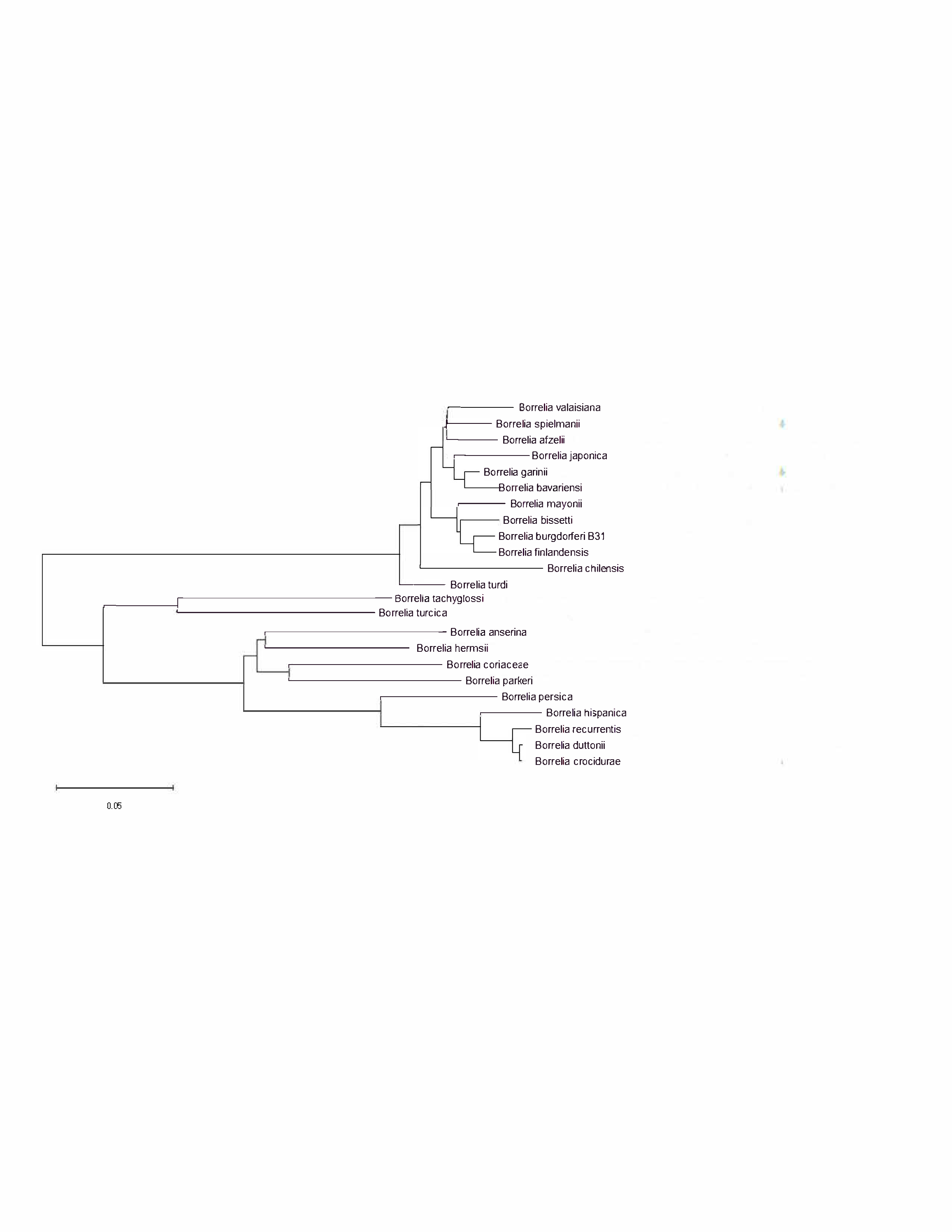

Supplement: S1 Fig — Evolutionary analyses were conducted in MEGA X using the Minimum Evolution method [1]. The analysis used 23 amino acid sequences and a total of 359 positions in the final dataset. The tree shown has a sum of branch length of 1.25967424. The branch lengths represent the units of evolutionary distances that infer the phylogenetic relationship calculated using the Poisson correction method. Distances indicate the number of amino acid substitutions per site. The Close-Neighbor-Interchange (CNI) algorithm was used to search the ME tree at a search level of 1. (TIFF) [file pone.0252214.s001.tiff]

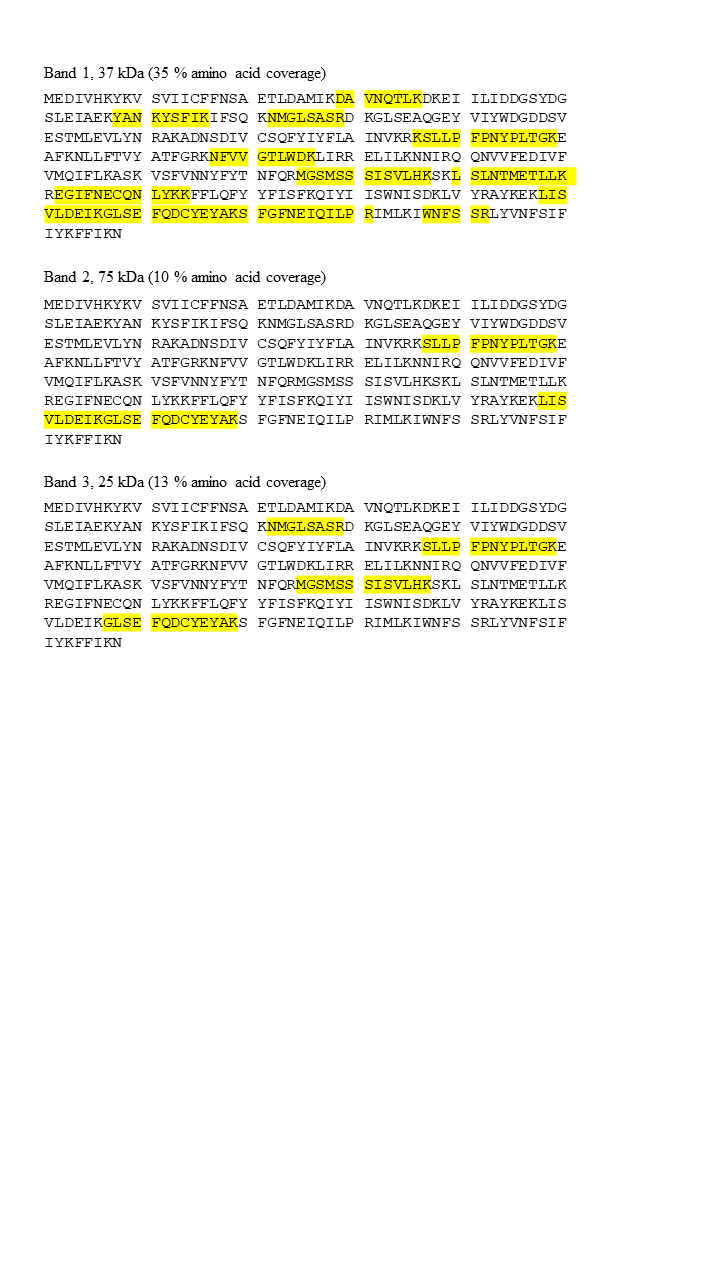

Supplement: S2 Fig — LC-MS/MS was performed on tryptic digests of gel bands at 37, 75 and 25 kDa bands observed by SDS-PAGE and Western blotting. The amino acid sequence shown is for BB0572 and the yellow highlighted regions represent the peptide sequences identified by LC-MS/MS. (TIF) [file pone.0252214.s002.tif]
