# Supplementary material for: Identification and functional analysis of a galactosyltransferase capable of cholesterol glycolipid formation in the Lyme disease spirochete Borrelia burgdorferi
Source: PLoS One. 2021 Jun 1;16(6):e0252214. doi: 10.1371/journal.pone.0252214 (PMC8168883; doi:10.1371/journal.pone.0252214)
Supplement: S1 File — (PDF) [file pone.0252214.s003.pdf]

PSORTb Results (Click here for an explanation of the output formats)

```
SeqID: WP_002665235.1 glycosyltransferase family 2 protein [Borreliella burgdorferi]
  Analysis Report:
    CMSVM-            Unknown                        [No details]
    CytoSVM-          Unknown                        [No details]
    ECSVM-            Unknown                        [No details]
    ModHMM-           Unknown                        [No internal helices found]
    Motif-            Unknown                        [No motifs found]
    OMPMotif-         Unknown                        [No motifs found]
    OMSVM-            Unknown                        [No details]
    PPSVM-            Unknown                        [No details]
    Profile-          Unknown                        [No matches to profiles found]
    SCL-BLAST-        CytoplasmicMembrane            [matched 62900119: Hyaluronan synthase]
    SCL-BLASTe-       Unknown                        [No matches against database]
    Signal-           Unknown                        [No signal peptide detected]
  Localization Scores:
    Cytoplasmic            0.15
    CytoplasmicMembrane    9.82
    Periplasmic            0.01
    OuterMembrane          0.01
    Extracellular          0.01
  Final Prediction:
    CytoplasmicMembrane    9.82
-------------------------------------------------------------------------------
```
