# Supplementary material for: Identification and functional analysis of a galactosyltransferase capable of cholesterol glycolipid formation in the Lyme disease spirochete Borrelia burgdorferi
Source: PLoS One. 2021 Jun 1;16(6):e0252214. doi: 10.1371/journal.pone.0252214 (PMC8168883; doi:10.1371/journal.pone.0252214)
Supplement: S4 File — (PDF) [file pone.0252214.s006.pdf]

Average Ct values for *flaB* housekeeping gene

Time of analysis	Ct values	Average Ct values	Ct values	Average Ct values	Standard deviation
	1[a]		2[a]		
10^6 base	14.4		14.68		
	13	13.7	13.38	14.03	0.233
6hrs	14.12		14.43		
	13.07	13.595	13.6	14.015	0.297
12hrs	13.95		12.98		
	13.58	13.765	11.82	12.4	0.965
18hrs	15.39		13.16		
	14.2	14.795	13.04	13.1	1.199
24hrs	11.96		11.38		
	12.67	12.315	11.48	11.43	0.626
30hrs	13.3		11.95		
	11.99	12.645	11.41	11.68	0.682
36hrs	12.91		12.74		
	12.82	12.865	12.75	12.745	0.085

[a] Biological replicate

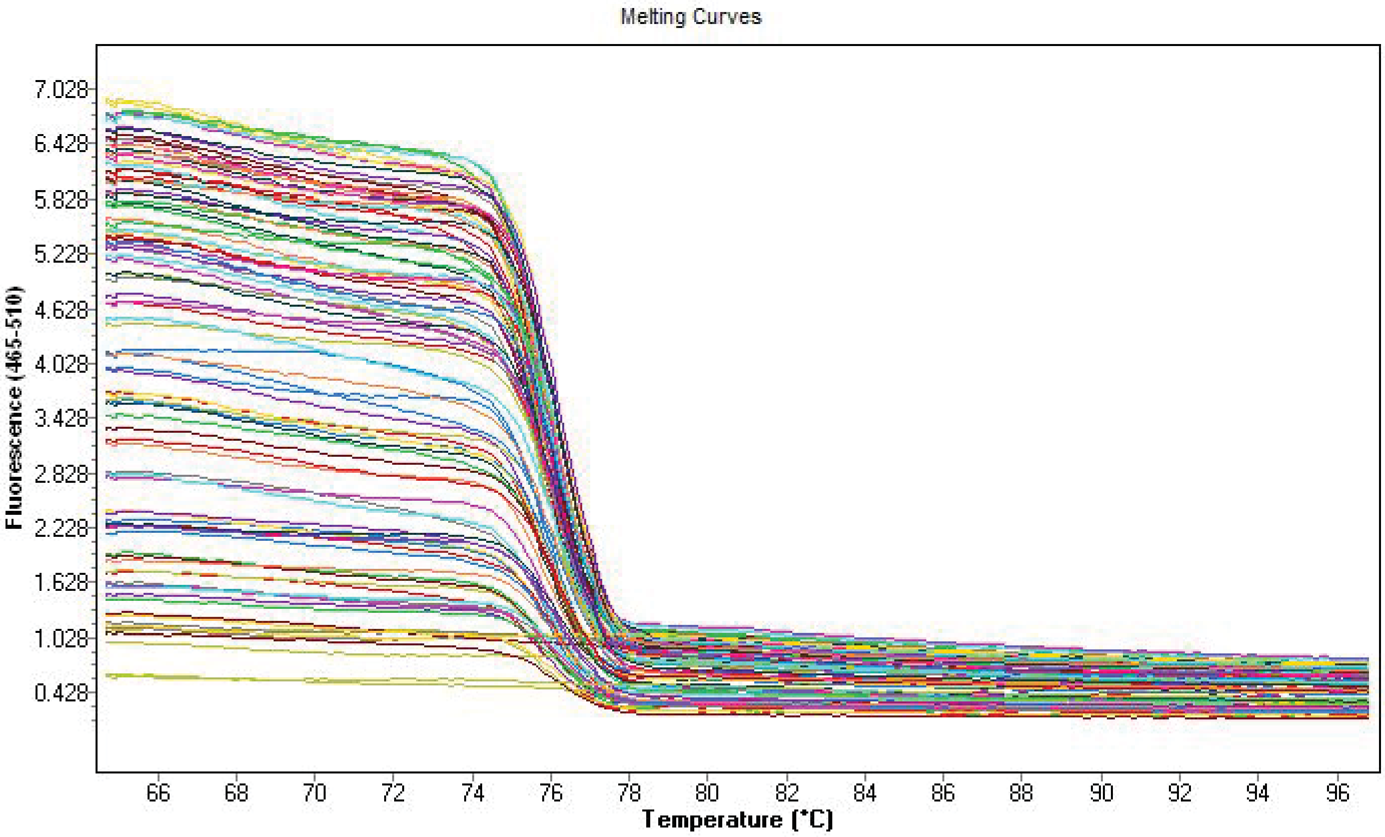

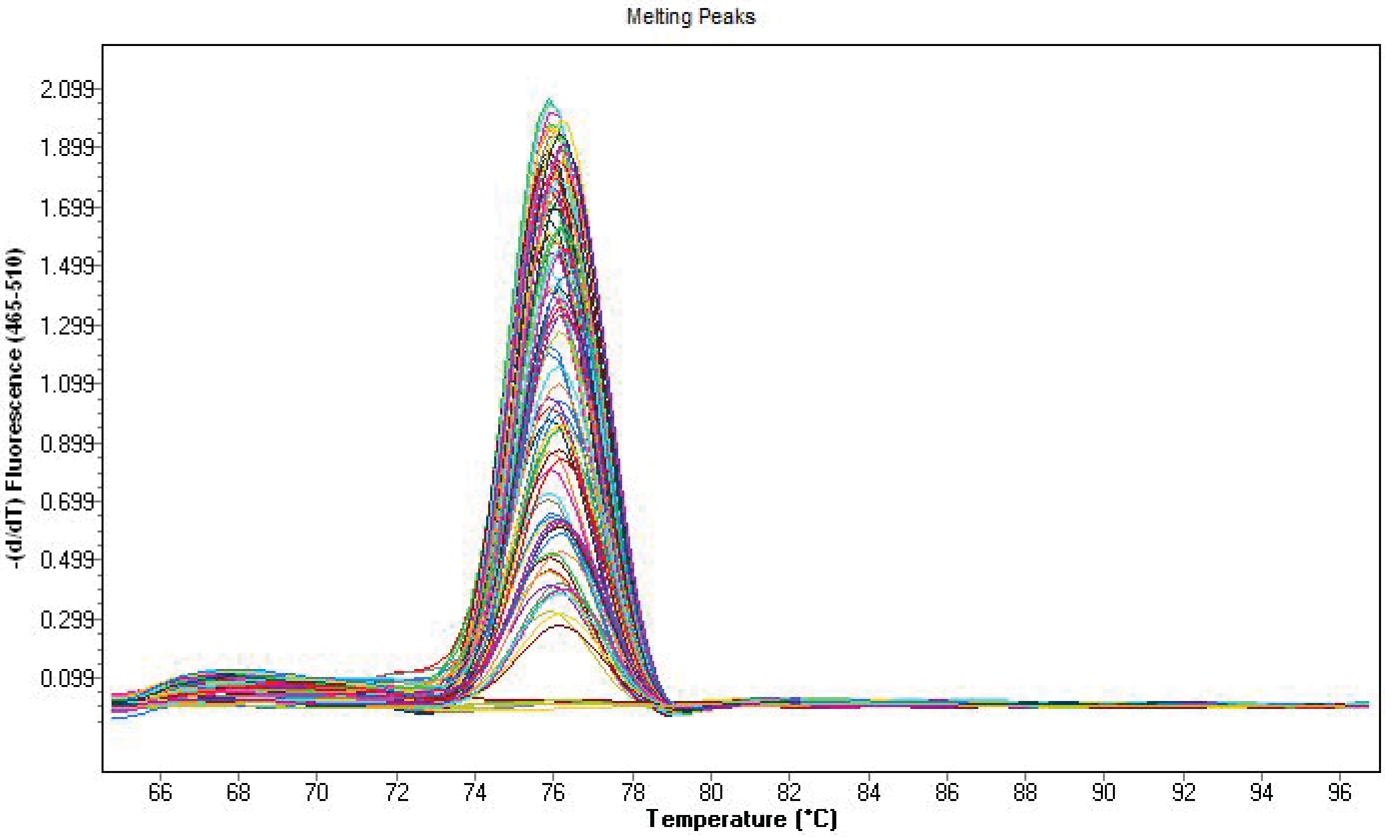

**Analysis Notes**	
