# Supplementary material for: Identification and functional analysis of a galactosyltransferase capable of cholesterol glycolipid formation in the Lyme disease spirochete Borrelia burgdorferi
Source: PLoS One. 2021 Jun 1;16(6):e0252214. doi: 10.1371/journal.pone.0252214 (PMC8168883; doi:10.1371/journal.pone.0252214)

Fig 3A

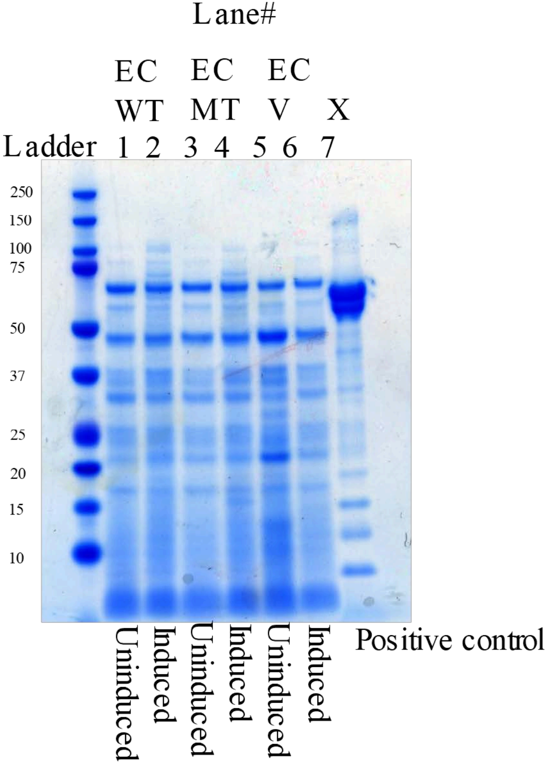

EC WT- *E coli* WT BB0572
EC MT- *E coli* Mutant BB0572
EC V- *E coli* empty vector
X- not included in main text

Fig 3 B

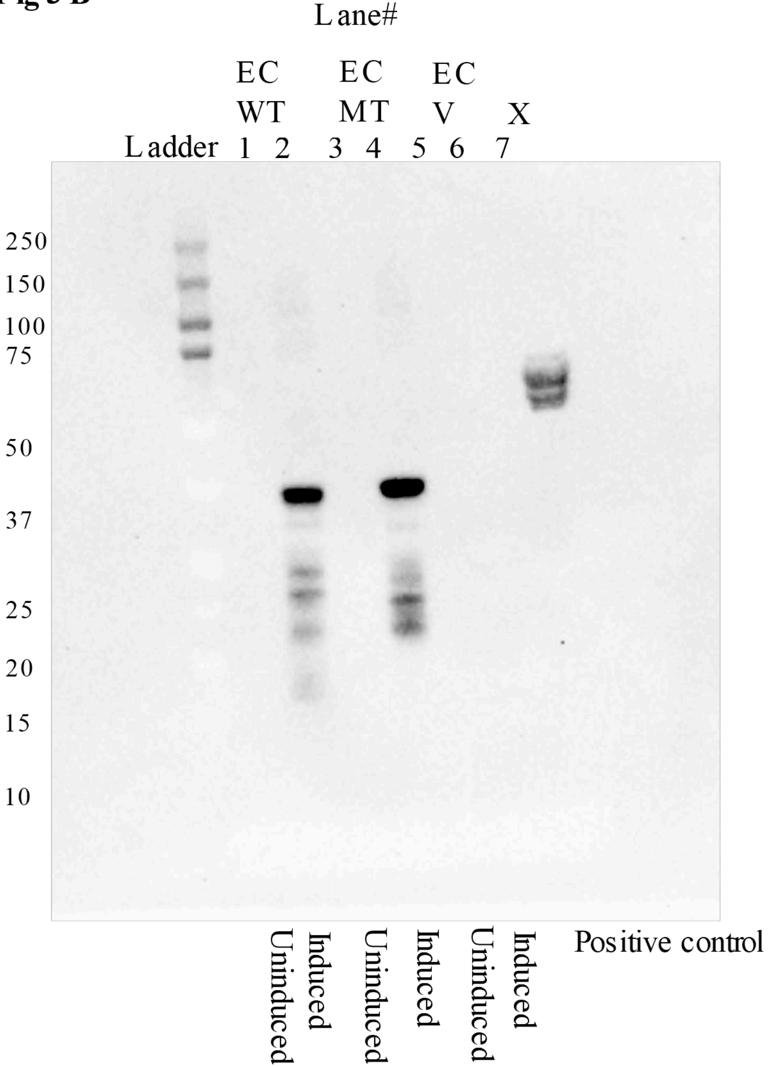

EC WT- *E coli* WT BB0572

EC MT- *E coli* Mutant BB0572

EC V- *E coli* empty vector

X- not included in main text

Fig 5. Undeveloped TLC blot and developed reference blot with non radioactive standards

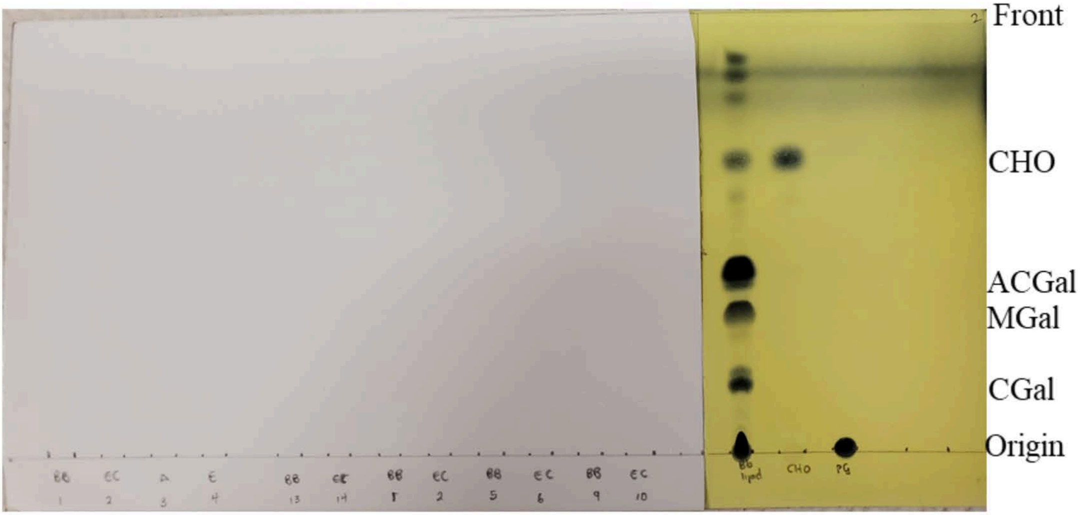

Developed TLC blot with marked references

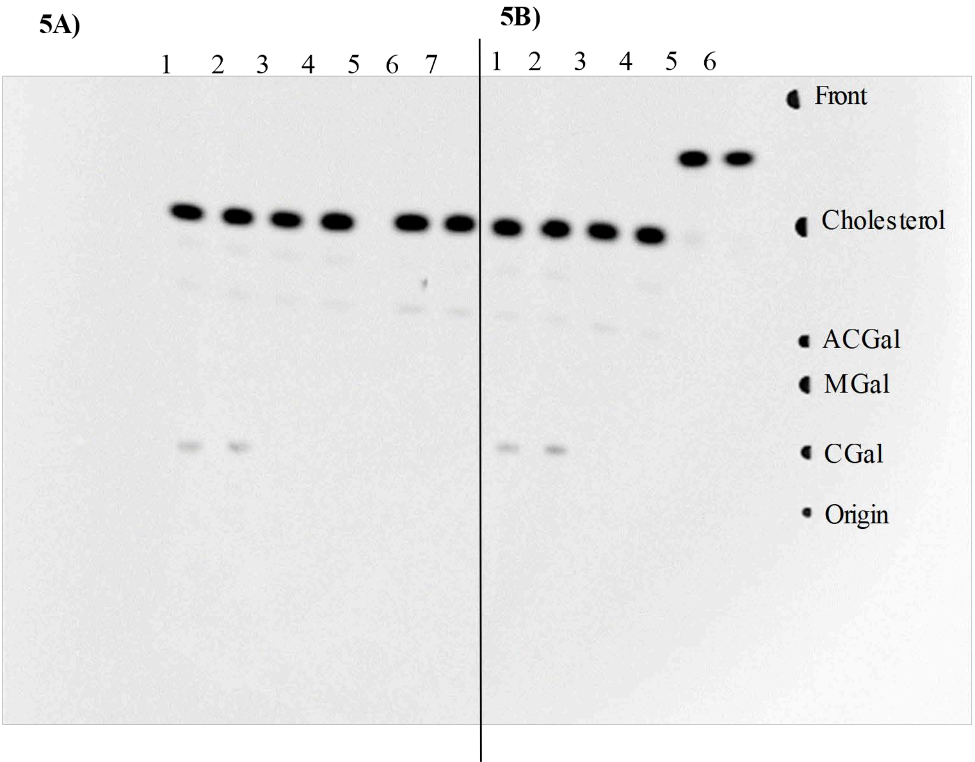

Supplement: S5 File — (PDF) [file pone.0252214.s007.pdf]
