# Supplementary material for: Identification and functional analysis of a galactosyltransferase capable of cholesterol glycolipid formation in the Lyme disease spirochete Borrelia burgdorferi
Source: PLoS One. 2021 Jun 1;16(6):e0252214. doi: 10.1371/journal.pone.0252214 (PMC8168883; doi:10.1371/journal.pone.0252214)
Supplement: S6 File — (DOCX) [file pone.0252214.s008.docx]

**Mass Spectrometry Analysis**

Samples derived from SDS-PAGE in-gel trypsin digestion and subsequent iodoacetamide alkylation were submitted for protein identification via LC-MS/MS to the Colorado State University Bioanalysis and Omics (ARC-BIO) Facility. Peptide extracts were resuspended in 5% ACN/0.1% formic acid. Once resolubilized, absorbance at 205 nm was measured on a NanoDrop (Thermo Fisher Scientific) and total peptide concentration was subsequently calculated using an extinction coefficient of 31 [1]. Reverse phase chromatography was performed using mobile phase consisted of water with 0.1% formic acid (A) and acetonitrile with 0.1% formic acid (B). A total of 0.5 μg of peptides were purified and concentrated using an on-line enrichment column (Waters Symmetry Trap C18 100 Å, 5 μm, 180 μm ID x 20 mm ) at 3%B. Subsequent chromatographic separation was performed on a Waters, Peptide BEH C18; 1.7 μm, 75 μm ID x 150 mm analytical column at 45°C using a 30 min gradient: 3 %-8 % buffer B over 3 minutes followed by 8%-35%B over 27 minutes at a flow rate of 350 nanoliter/min. Peptides were introduced directly into the mass spectrometer (Orbitrap Velos Pro, Thermo Fisher Scientific) equipped with a Nanospray Flex ion source (Thermo Fisher Scientific) and spectra were collected over a *m*/*z* range of 400–2000, positive mode ionization. Ions with charge state +2 or +3 were accepted for MS/MS using a dynamic exclusion limit of 2 MS/MS spectra of a given *m*/*z* value for 30 s (exclusion duration of 90 s). The instrument was operated in FT mode for MS detection (resolution of 60,000) and ion trap mode for MS/MS detection with a normalized collision energy set to 35%. Compound lists of the resulting spectra were generated using Xcalibur 3.0 software (Thermo Fisher Scientific) with a S/N threshold of 1.5 and 1 scan/group.

**Data Analysis**

LC-MS/MS data were extracted, charge state deconvoluted and deisotoped by ProteoWizard MsConvert (version 3.0). Spectra from all samples were searched using Mascot (Matrix Science, London, UK; version 2.6.0) against the BB0572 protein sequence plus the Uniprot reference proteome for *E. coli* strain K12 (UP000000625; downloaded 22 February 2018) and a database of common contaminants (cRAP; downloaded 5 October 2018) assuming the digestion enzyme trypsin. Mascot was searched with a fragment ion mass tolerance of 0.80 Da and a precursor ion tolerance of 20 PPM. Carbamidomethyl of cysteine was specified in Mascot as a fixed modification. Deamidation of asparagine and glutamine and oxidation of methionine were specified in Mascot as variable modifications.

Search results from all samples were imported and combined using the probabilistic protein identification algorithms [2] implemented in the Scaffold software (version Scaffold_4.9.0, Proteome Software Inc., Portland, OR) [3]. Peptide thresholds were set (90%) such that a peptide FDR of 0.0% was achieved based on hits to the reverse database [4]. Protein identifications were accepted if they could be established at greater than 95.0% probability and contained at least 2 identified peptides. Protein probabilities were assigned by the Protein Prophet algorithm [5]. Proteins that contained similar peptides and could not be differentiated based on MS/MS analysis alone were grouped to satisfy the principles of parsimony.

**References**

1. Scopes RK. Measurement of protein by spectrophotometry at 205 nm. Analytical biochemistry. 1974;59(1):277-82.

2. Keller A, Nesvizhskii AI, Kolker E, Aebersold R. Empirical statistical model to estimate the accuracy of peptide identifications made by MS/MS and database search. Analytical chemistry. 2002;74(20):5383-92.

3. Searle BC, Turner M, Nesvizhskii AI. Improving sensitivity by probabilistically combining results from multiple MS/MS search methodologies. J Proteome Res. 2008;7(1):245-53.

4. Käll L, Storey JD, MacCoss MJ, Noble WS. Assigning significance to peptides identified by tandem mass spectrometry using decoy databases. J Proteome Res. 2008;7(1):29-34.

5. Nesvizhskii AI, Keller A, Kolker E, Aebersold R. A statistical model for identifying proteins by tandem mass spectrometry. Analytical chemistry. 2003;75(17):4646-58.
