# Supplementary material for: Identification and functional analysis of a galactosyltransferase capable of cholesterol glycolipid formation in the Lyme disease spirochete Borrelia burgdorferi
Source: PLoS One. 2021 Jun 1;16(6):e0252214. doi: 10.1371/journal.pone.0252214 (PMC8168883; doi:10.1371/journal.pone.0252214)
Supplement: S1 Table — (DOCX) [file pone.0252214.s009.docx]

**Table S1. Selected *Borrelia* species for protein alignment and evolutionary analyses**

| **Species name** | **NCBI Reference Sequence:**  **Glycosyltransferase family 2 protein** |
| --- | --- |
| **Lyme disease causing spirochetes** |  |
| *Borrelia burgdorferi* | AAC66931.1 |
| *Borrelia finlandensis* | WP_008882777.1 |
| *Borrelia bissettii* | WP_071983512.1 |
| *Borrelia mayonii* | WP_075552234.1 |
| *Borrelia valaisiana* | WP_006068751.1 |
| *Borrelia garinii* | WP_004791126.1 |
| *Borrelia spielmanii* | WP_006433876.1 |
| *Borrelia afzelii* | WP_015055652.1 |
| *Borrelia bavariensis* | WP_044007903.1 |
| *Borrelia japonica* | WP_091971997.1 |
| *Borrelia turdi* | WP_151060205.1 |
| *Borrelia chilensis* | AJA90372.1 |
| **Relapsing fever causing spirochetes** |  |
| *Borrelia tachyglossi* | WP_108729338.1 |
| *Borrelia anserina* | WP_025419740.1 |
| *Borrelia persica* | WP_024653465.1 |
| *Borrelia turcica* | WP_120104355.1 |
| *Borrelia coriaceae* | WP_038364640.1 |
| *Borrelia hermsii* | WP_025406623.1 |
| *Borrelia parkeri* | WP_038447867.1 |
| *Borrelia duttonii* | WP_012538322.1 |
| *Borrelia crocidurae* | WP_014696404.1 |
| *Borrelia recurrentis* | WP_012539002.1 |
| *Borrelia hispanica* | WP_024654841.1 |
